# Human–Bat Interactions in Rural West Africa

**DOI:** 10.3201/eid2108.142015

**Published:** 2015-08

**Authors:** Priscilla Anti, Michael Owusu, Olivia Agbenyega, Augustina Annan, Ebenezer Kofi Badu, Evans Ewald Nkrumah, Marco Tschapka, Samuel Oppong, Yaw Adu-Sarkodie, Christian Drosten

**Affiliations:** Kwame Nkrumah University of Science and Technology, Kumasi, Ghana (P. Anti, M. Owusu, O. Agbenyega, A. Annan, E.K. Badu, E.E. Nkrumah, S. Oppong, Y. Adu-Sarkodie);; University of Ulm, Ulm, Germany (M. Tschapka);; University of Bonn Medical Centre, Bonn, Germany (C. Drosten);; German Centre for Infection Research, Bonn (C. Drosten)

**Keywords:** bats, virus reservoir, Ebola, SARS, severe acute respiratory syndrome, MERS, Middle East respiratory syndrome, Nipah, viruses, West Africa, zoonoses

## Abstract

Because some bats host viruses with zoonotic potential, we investigated human–bat interactions in rural Ghana during 2011–2012. Nearly half (46.6%) of respondents regularly visited bat caves; 37.4% had been bitten, scratched, or exposed to bat urine; and 45.6% ate bat meat. Human–bat interactions in rural Ghana are frequent and diverse.

Bats are increasingly being recognized as hosts for pathogens that affect humans and livestock (*1*). The 2014–2015 outbreak of Ebola virus disease in West Africa demonstrates how human–bat interactions in even remote locations can trigger infection chains that affect global public health and strain the national health care systems in Africa (*2*). One of the major challenges to preventing bat-related diseases is lack of knowledge about the frequency of, circumstances surrounding, and motivations for human–bat interactions in rural African communities. Only a few quantitative records are available in the scientific literature, and most are not specific for Africa (*3*). 

In Ghana, bats carry potentially zoonotic viruses including lyssa-, corona-, henipa-, and filoviruses (*4*–*6*). Although anecdotal knowledge exists with regard to human contact with bats and bat roosts within rural communities and information about the ubiquitous bush meat trade (*7*), little information is available about the intensity and circumstances of exposure (*8*). We therefore studied the cultural practices, sociodemographic factors, and religious activities that determine human–bat contact in remote rural communities from which new disease outbreaks have repeatedly emerged (*9*). Specifically, we studied the sociocultural association of humans with bats in rural communities in Ghana, focusing on potential routes of virus transmission.

## The Study

**Figure 1:**
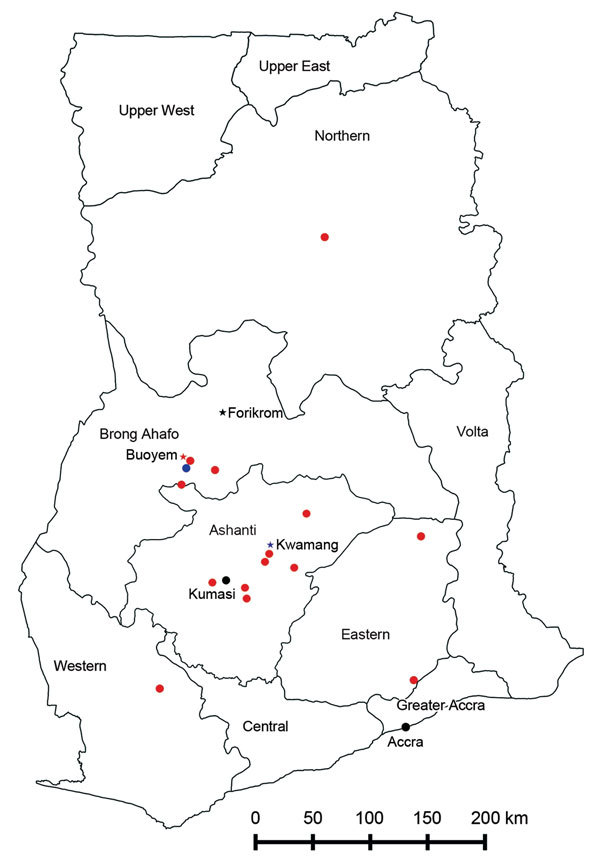
Human–bat interaction study locations and provinces within Ghana, 2011–2012. Asterisks indicate the study sites, Kwamang, Forikrom, and Buoyem. Red circles indicate sources of bush meat. The main Techiman market is situated in the Techiman municipality (blue circle); this market is ≈15 km from Buoyem and is the largest and most economically active market in the Brong Ahafo region. Accra and Kumasi, the largest cities in Ghana, also receive supplies of bat meat from the Techiman market.

The study was conducted in 3 communities in Ghana: Kwamang (population 8,000), Forikrom (population 3,800), and Buoyem (population 3,900). Kwamang is part of the Ashanti Province; Buoyem and Forikrom are in Brong Ahafo Province (Figure 1). Ethics approval was obtained from the Committee for Human Research, Publications and Ethics of Komfo Anokye Teaching Hospital and School of Medical Sciences, Kwame Nkrumah University of Science and Technology, Kumasi.

In each of the 3 communities, in-depth interviews of local leaders were conducted. Buoyem leaders described an activity called the Yam Festival, a hunting festival during which men took ladders to caves on Wednesday evenings and caught bats as they returned from feeding. These bats were described as fruit bats and thus were possibly *Rousettus aegyptiacus* bats, the species most commonly identified in Buoyem caves. The night’s catch was collected by the women; menstruating women were excluded from participation in Yam activities for reasons explained as cleanliness. In recent years, Yam activities had been discontinued because of chieftaincy disputes and conflict over ownership of cave lands. Traditional authorities in Kwamang and Forikrom did not report similar cultural activities in connection with bats.

**Figure 2:**
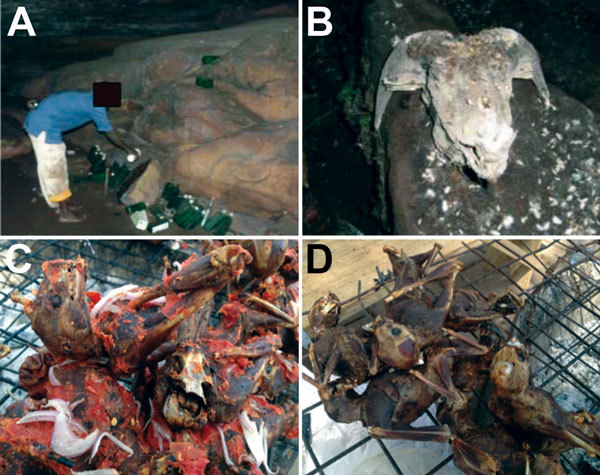
Typical situations in which direct and indirect bat–human contact occurred in Ghana, 2011–2012. A) Religious activity at the Mprisi cave in Buoyem. The man is pouring libation to the natural gods. The liquid poured before entering the cave is liquor. Note the number of deposited empty bottles, indicating the frequency of cave entries. B) Goat sacrificed for natural gods at the Mframmabuom cave in Kwamang. C, D) Typical examples of roasted bats widely offered and consumed in markets and public places in Ghana. Photographs provided by and published with permission from H. Baldwin.

Regular human activities were directly observed at all cave sites, including the Mprisi (Figure 2, panel A) and Dwamerewa caves in Bouyem, Boten cave in Forikrom, and Mmframabuom and Ohene Abutia caves in Kwamang (Figure 2, panel B). The Ohene Abutia cave served as one of the major water sources in the Kwamang community. Several caves served as spiritual sanctuaries. Focus group discussions were conducted in all communities (Technical Appendix).

Structured household survey questionnaires were received back from 1,274 respondents: 32.3% from Buoyem, 28.4% from Forikrom, and 39.2% from Kwamang. Contact with bats was reported by 841 (66%) respondents; bat bites, scratches, or urine exposure was reported by 476 (37.4%) respondents. Almost half (594 [46.6%]) of respondents visited bat caves frequently; 217 (17%) reported coming into contact with bats only in their normal living or work environment (Table). The proportion of respondents who deliberately visited caves was significantly higher than the proportion exposed only in their living and work environments (p<0.001).

**Table:** Modes of human–bat contact and purposes of cave visitation, Ghana, 2011–2012*

Contact	Community, no. (%)		
	Buoyem, n = 412	Forikrom, n = 362	Kwamang, n = 500
Respondents reporting bat contact	263 (63.8)	244 (67.4)	334 (66.8)
In houses through broken ceilings	69 (16.7)	51 (14.1)	65 (13)
In bat roosts on farms	41 (10)	28 (7.7)	63 (12.6)
In caves	129 (31.3)	161 (44.5)	187 (37.5)
At work places	0	1 (0.3)	0
In school buildings	24 (5.8)	3 (0.8)	5 (1)
In other areas	0	0	14 (2.8)
Respondents visiting bat caves	181 (43.9)	178 (49.3)	222 (44.4)
For religious activities	19 (4.6)	79 (21.8)	5 (1)
For recreation	58 (14.1)	73 (20.2)	46 (9.2)
To collect bat guano	0	14 (3.9)	2 (0.4)
To fetch water	1 (0.2)	0	123 (24.6)
To hunt for bats	102 (24.8)	6 (1.7)	10 (2)
To farm	9 (2.2)	17 (4.7)	33 (6.6)
For other reasons	2(0.5)	5 (1.4)	14 (2.8)

*Data based on focus group discussions and stratified household surveys (Technical Appendix).

Bat species identification was based on observations and standard illustrated field guides (*10*). Focus group participants identified bats species by using standard images of species recorded from each study site. Observed insectivorous bats included *Nycteris* spp. (Nycteriidae), *Hipposideros jonesi, H.* aff. *Ruber*, *H. gigas*, and *H. abae* (Hipposideridae); observed fruit bats included *Hypsignathus monstrosus*, *Rousettus aegyptiacus*, and *Eidolon helvum* (Pteropodidae). These bat species are known to carry coronaviruses (particularly Hipposideridae bats) (*11*); hantaviruses (particularly Nycteridae bats) (*12*); paramyxoviruses, including henipavirus (*13*); and filoviruses (*14*).

Trading of roasted and fried bats was widely observed in market places (Figure 2, panel C, and Figure 1, panel D). Initial information about the supply routes of bat meat obtained from hunters and members of the indigenous community led to investigation of the bat meat trade at the main market in Techiman. Hunters from the surrounding communities supplied most traded bats. Information gathered from traders showed that the supply route of bat meat extends far beyond the Brong Ahafo region to other regions in Ghana and neighboring countries (Figure 1). Some places mentioned by the traders as sources of bat meat include towns and villages in the Ashanti region. Some of these were Duamo (3 km from Kwamang), Adobomam, Kyekyebon, Kumawu, Deduako, Agogo, and the zoological gardens in Kumasi, where migratory *E. helvum* bats roost seasonally (*13*). Other areas were in Techiman, Nkoranza, Tanoso, and Tuobodom in the Brong Ahafo region; Afram Plains and Akuapem in the Eastern region, and Accra in the Greater Accra region. Some supplies came from the Northern region and beyond the borders of Ghana from Côte d’Ivoire.

Of the 1,274 respondents, 581 (45.6%) reported having consumed bats. Among these, 257 (44.2%) respondents were from Buoyem, 141 (24.2%) from Forikrom, and 183 (31.5%) from Kwamang (Technical Appendix Table 1). Of the 581 respondents who ate bat meat, 237 (40.8%) obtained bats from caves, 123 (21.1%) caught bats on farms with bat roosts, 114 (19.6) bought bats from community markets, and 60 (10.3%) bought bats from restaurants as part of meals served. Most respondents described the consumed animals as “big bats,” suggesting that most were fruit bats (Pteropodidae).

To identify the factors associated with bat consumption, we compared determinant variables for the 581 respondents who consumed bats and the 690 who did not (Technical Appendix Table 2). Bat meat was eaten by a significantly higher percentage of men than women (p<0.001) and a significantly higher proportion of farmers than those with other occupations (p<0.001). To determine the variables that significantly influenced the consumption of bat meat, we entered all significant variables into a logistic regression model. The odds of consuming bat meat were higher for men (odds ratio 2.47; 95% CI 1.93–3.17) than for women and for respondents >25 years of age (odds ratio 4.14; 95% CI 2.91–5.89) than for those <25 years of age (Technical Appendix Table 3).

A second multivariate analysis, conducted to determine factors that predict visitation of bat caves, indicated that older age and male sex were significantly associated with visitation of bat caves (Technical Appendix). The association between cave visitation and bat consumption was significant (χ^2^ = 75.6; p<0.001); odds of eating bat meat were twice as high among respondents who visited bat caves (odds ratio 2.74) than among those who did not.

## Conclusions

The deliberate entry into bat caves represents a prevalent behavior that could be influenced by community-level education in the aftermath of the ongoing outbreak of Ebola virus disease in West Africa. Another obvious target is the widespread bat meat trade and consumption. Further research will be necessary for understanding belief systems and developing acceptable guidance for rural communities exposed to bats because of traditional and spiritual reasons.

**Technical Appendix.** Supplemental materials and methods; sources and processing of bat meat in Ghana; factors associated with consumption of bat meat; and predictors of bat meat consumption and bat cave visitation.
