# Supplementary material for: Human–Bat Interactions in Rural West Africa
Source: Emerg Infect Dis. 2015 Aug;21(8):1418–21. doi: 10.3201/eid2108.142015 (PMC4517717; doi:10.3201/eid2108.142015)
Supplement: Supplementary file 1 — Technical Appendix. Supplemental materials and methods; sources and processing of bat meat in Ghana; factors associated with consumption of bat meat; and predictors of bat meat consumption and bat cave visitation. [file 14-2015-Techapp-s1.pdf]

Article DOI: http://dx.doi.org/10.3201/eid2108.142015

Human–Bat Interactions in Rural West Africa

Technical Appendix

Supplemental Materials and Methods

Focus group discussions

Focus group discussions were organized in each of the study areas to assess the knowledge and cultural beliefs of subjects on bats (*1*). To ensure unbiased representation from the community, subjects with diverse occupations and economic status were selected. Discussion guides were used to generate open discussions in three key areas: importance of bats, cultural beliefs about bats and negative impacts of bats in the communities. Subjects were organized in 8–12 per group and interviews were conducted by trained field research assistants. General discussions especially on sensitive subjects was led by a group leader selected by participants. The focus group leader concept was used to encourage participants to disclose information freely. Responses obtained from the groups were documented and similar results were aggregated using tallies. All discussions were conducted in the local dialect of respondents.

Stratified random sampling for household surveys

The study site was divided into four areas based on the major roads. Social centers in each quadrant were identified, and every other adjacent house starting from the social center was marked and respondents were interviewed using structured questionnaires. Respondents were from 13 years and above.

Data management and statistical analysis methods

Data was collected on demographics, bat cave-associated activities, contact with bats, bites and scratches from bats and the use of bats for food, medicinal or ritual purposes. An exposure to bats was defined as a bite or scratch from a bat or circumstances such as direct skin contact with a bat, bat urine or guano.

Quantitative data from questionnaires were recorded using EPI INFO version 5 (*2*), and imported into Microsoft® Excel. Subsequent analysis was performed using R statistical software version 3.0.2 (*3*). Categorical variables were analyzed using Chi-square or Fischer's

exact test depending on the cell sample sizes being compared. Continuous variables were expressed as medians with their inter-quartile ranges (IQR).

The associated factors influencing the consumption of bat meat and visitation of caves were assessed by entering all variables that were significant at $p < 0.1$ from the bivariate analysis into an unconditional multiple logistic regression model. A backward stepwise approach was used for selection of significant variables from the model. All results were expressed as adjusted odd ratios and 95% confidence interval (CI). For all analysis, a two-sided p-value of less than 0.05 was considered significant.

Technical Appendix Table 1. Sources and processing of bat meat

**Community**	**Buoyem**	**Forikrom**	**Kwamang**		**TEST***
Respondents	n = 257	n = 141	n = 183	Tests	P-value
**Sources of bat meat**	n (%)	n (%)	n (%)		
Bat Caves	179 (69.6)	10 (7.1)	48 (26.2)	X^2 (2df) = 171	**<0.001**
Markets	22 (8.6)	23 (16.3)	69 (37.7)	X^2 (2df) = 58.9	**<0.001**
Bat roost in farms	18 (7)	61 (43.3)	44 (24)	X^2 (2df) = 73.1	**<0.001**
Homes	8 (3.1)	15 (10.6)	3 (1.6)	X^2 (2df) = 17.1	**<0.001**
Hunters	28 (10.9)	14 (9.9)	3 (1.6)	X^2 (2df) = 14.1	**0.001**
Restaurants/Food vendors	25 (9.7)	17 (12.1)	18 (9.8)	X^2 (2df) = 0.6	0.74
Other sources of bat	2 (0.8)	1 (0.7)	7 (3.8)	Fisher's exact	0.046
**Respondents mode of catching bats**	n (%)	n (%)	n (%)		
Hands	134 (52.1)	1 (0.7)	2 (1.1)	X^2 (2df) = 208.6	**<0.001**
Guns	15 (5.8)	25 (17.7)	56 (30.6)	X^2 (2df) = 47.7	**<0.001**
Net	2 (0.8)	20 (14.2)	0	X^2 (2df) = 55.4	**<0.001**
Sticks	54 (21)	4 (2.8)	12 (6.6)	X^2 (2df) = 36.0	**<0.001**
Catapult	18 (7)	31 (22)	18 (9.8)	X^2 (2df) = 20.8	**<0.001**
Other Mode of capture	1 (0.4)	5 (3.5)	8 (4.4)	Fisher's exact	**0.004**
**Type of bat consumed**	n (%)	n (%)	n (%)	Fisher's exact	0.991
Big bats	250 (97.3)	137 (97.2)	177 (96.7)		
Small bats	1 (0.4)	1 (0.7)	1 (0.5)		
Not known	6 (2.3)	3 (2.1)	5 (2.7)		
**Mode of cooking bats**	n (%)	n (%)	n (%)		
Boiling	201 (78.1)	128 (90.8)	120 (65.6)	**X^2 (2df) = 29.1**	**<0.001**
Roasting	217 (84.4)	64 (45.4)	144 (78.7)	**X^2 (2df) = 74.9**	**<0.001**
Frying	2 (0.8)	0	0	Fisher's exact	0.518
Other modes of bat processing	2 (0.8)	1 (0.7)	0	Fisher's exact	0.603

The proportions of respondents who reported either Yes or No for each categorical variable were compared across the three communities using Fischer's exact test or Chi-square test (X^2) where appropriate. A p-value of less than 0.05 was considered statistically significant. All p-values less than 0.001 were abbreviated to "< 0.001". "df" denotes degrees of freedom.

Technical Appendix Table 2. Factors associated with consumption of bat meat

	Not Consumed bats	Consumed bats	Test	P-value
	n = 690	n = 581		
Variables	n (%)	n (%)		
Gender Male	245 (35.5)	309 (53.2)	X^2 (1 df) = 39.4	**<0.001**
Gender Male	245 (35.5)	309 (53.2)	X^2 (1 df) = 39.4	**<0.001**
Highest level of education - Primary Education	100 (14.5)	69 (11.9)	X^2 (1 df) = 1.7	0.199
Highest level of education -JHS Education	192 (27.8)	134 (23.1)	X^2 (1 df) = 3.5	0.061
Highest level of education - SHS Education	210 (30.4)	179 (30.8)	X^2 (1 df) = 0.01	0.934
Highest level of education - Tertiary Education	25 (3.6)	20 (3.4)	X^2 (1 df) <0.001	0.983
Christians	640 (92.8)	531 (91.4)	X^2 (1 df) = 0.6	0.428
Muslims	30 (4.3)	6 (1)	X^2 (1 df) = 11.4	**<0.001**
Traditionalists	4 (0.6)	5 (0.9)	X^2 (1 df) = 0.07	0.796
Belonging to other religions	14 (2)	34 (5.9)	X^2 (1 df) = 11.7	**<0.001**
Age groups of respondents			X^2 (2 df) = 137.5	**<0.001**
10 - 25	305 (44.6)	90 (15.6)		
26 - 45	195 (28.5)	187 (32.4)		
46 - 115	184 (26.9)	301 (52.1)		
Students	191 (27.7)	52 (9)	X^2 (1 df) = 70.4	**<0.001**
Farmers	226 (32.8)	359 (61.8)	X^2 (1 df) = 105.9	**<0.001**
Teachers	11 (1.6)	21 (3.6)	X^2 (1 df) = 4.5	**0.035**

The proportions of respondents who reported either Yes or No for each categorical variable were compared between those who consumed bats and those who did not using Fischer's exact test or chi-square test where appropriate. A p-value of less than 0.05 was considered statistically significant. All p-values less than 0.001 were abbreviated as "<0.001." JHS: Junior High School; SHS: Senior High School and df = degrees of freedom.

Technical Appendix Table 3. Final multivariate model

**Predictors of bat consumption**				
Variables	Crude OR (95% CI)	Adj OR (95% CI)	P (Wald's test)	P (LR-test)
Age Group ref (10 - 25)				<0.001
26 - 45	3.25 (2.39,4.43)	2.79 (1.98, 3.91)	<0.001	
46 - 115	5.54 (4.11, 7.47)	4.14 (2.91, 5.89)	<0.001	
Gender ref (Female)	2.04 (1.63, 2.56)	2.47 (1.93, 3.17)	<0.001	<0.001
Farming ref (Not Farming)	3.27 (2.6, 4.13)	1.93 (1.46, 2.55)	<0.001	<0.001
**Predictors of cave visitation**				
	Crude OR (95% CI)	Adj OR (95% CI)	P (Wald's test)	P (LR-test)
Age Group ref (10 - 25)				<0.001
26 - 45	1.5 (1.13, 2)	1.57 (1.17, 2.1)	0.003	
46 - 115	2.01 (1.53, 2.64)	2.08 (1.58, 2.74)	<0.001	
Gender ref (Female)	1.69 (1.35, 2.12)	1.74 (1.38, 2.19)	<0.001	<0.001

P-values less than 0.001 were abbreviated as "<0.001." Crude OR represents crude odds ratio, Adj OR represents adjusted odds ratio and LR represents likelihood ratio test.

The proportions of respondents who reported either yes or no for each categorical variable were compared across the three

References

1. Bryman A. 2012. Social research methods. OUP Oxford.

2. Centers for Disease Control and Prevention. 2007. Epi Info, v3.4.3.

3. R Development Core Team. 2013. A language and environment for statistical computing. R Foundation for Statistical Computing., Vienna, Austria, http://www.R-project.org.
